# Why the EMTALA Mandate for Emergency Care Does not Equal Healthcare “Coverage”

**DOI:** 10.5811/westjem.2017.5.34826

**Published:** 2017-05-15

**Authors:** Nicolas T. Sawyer

**Affiliations:** University of California, Davis, Department of Emergency Medicine, Davis, California

House GOP members Rep. Mark Meadows (R-NC) and Rep. Raúl Labrador (R-ID) recently made alarming and misleading statements about American emergency departments’ role in U.S. healthcare. In March of this year, while speaking with CNN’s New Day host Alisyn Camerota, Mr. Meadows stated:

“The goal is to allow access to all. There’s a federal law right now that if you show up at a hospital, you get coverage, Alisyn. And so, it’s a false narrative to suggest we have people who can’t go in and get coverage. It’s a federal law.”^1^

After passing the House GOP American Health Care Act (AHCA) last Thursday, which would allow individual states to seek waivers to eliminate essential health benefits, including emergency department visits, this narrative was reiterated by Mr. Labrador at a town hall meeting in Southern Idaho. The statement was met with loud boos by constituents, and a video of the event has been widely shared on social media. In response, Labrador stated on Saturday:

“In the five-second clip that the media is focusing on, I was trying to explain that all hospitals are required by law to treat patients in need to [sic] emergency care regardless of their ability to pay and that the Republican plan does not change that.”^2^

It is vital that all Americans, including Mr. Meadows and Mr. Labrador understand the details of the law they are referencing, including why the law certainly does not provide healthcare access or “coverage.” The law, called the Emergency Medical Treatment and Labor Act (EMTALA) was passed in 1986 in response to “patient dumping,” the practice of hospitals refusing to treat people with medical emergencies because of their inability to pay or insufficient insurance. “Patient dumping” also applies to early and inappropriate hospital discharge due to high anticipated treatment costs.

Firstly, EMTALA only applies to “participating hospitals,” those that accept Medicare and Medicaid payments. Combined payments for Medicare and Medicaid in 2015 totaled $1.19 trillion, making up 45% of national health expenditures, which total $3.2 trillion. This makes not participating in EMTALA impractical for nearly all hospitals.^3^ However, the law does not apply to doctors’ offices or clinics, so it has no effect on preventive or primary care.

Next, contrary to the misconception assumed by many, including Mr. Meadows and Labrador, EMTALA does not mandate treatment of non-emergent conditions. EMTALA only mandates that providers provide a “medical screening exam” including blood tests, imaging, and consultation with specialists as necessary to decide whether an emergency medical condition (EMC) does or does not exist. The U.S. government defines an EMC as “a condition manifesting itself by acute symptoms of sufficient severity (including severe pain) such that the absence of immediate medical attention could reasonably be expected to result in placing the individual’s health [or the health of an unborn child] in serious jeopardy, serious impairment to bodily functions, or serious dysfunction of bodily organs.”^4^ For example, a patient presenting with a heart attack must be treated by emergency physicians and interventional cardiologists until their blocked coronary artery is reopened. But it says nothing about the ongoing care of the heart patient, unless and until there’s another emergency. Nor does it “cover” any prevention to slow or mitigate the development of heart disease. How foolish is it to require treatment for the emergency only, and yet not “cover” any post-emergency care, or try to prevent the crisis in the first place?

While Mr. Meadows and Mr. Labrador are correct in saying that under EMTALA, Americans presenting to their local emergency departments are eligible to receive care, the law does not mandate care be provided under three caveats:

1. patients will only receive care if they have an EMC;
2. EMTALA contains no requirement for physicians and hospitals to provide uncompensated care or stabilizing treatment for patients with non-emergency conditions; and
3. uninsured or underinsured patients are still responsible for the costs of care and will be personally billed for all services. There is no “coverage” at all, only mandated emergency care for which the patient still must pay (or go bankrupt).

EMTALA was passed in 1986 without any funding whatsoever, so there is no “insurance” component to the law that our congressmen refer to as “coverage.” EMTALA is considered by many to be an “unfunded mandate.”^4^

Further, stating that access to care in emergency departments implies access to care in general ignores the fact that board-certified emergency physicians, like myself, are trained as experts in emergencies, not routine primary care. We spent years becoming experts in the diagnosis and treatment of life-threatening conditions such as cardiac arrest, respiratory failure, acute kidney failure, shock states (very low blood pressure), emergent child delivery, poisonings, acute heart failure, stroke, neonatal emergencies, blunt and penetrating injury, and much more.

We do not specialize in routine health maintenance including disease prevention or surveillance, and management of chronic diseases like high blood pressure, diabetes, asthma, heart disease, cancer, obesity, arthritis, chronic pain, psychiatric disorders, and a variety of other “pre-existing conditions” affecting millions. If the AHCA is signed into law in its current form, millions of Americans will once again be uninsured, preventing them from accessing primary care.^5^ Although the Congressional Budget Office estimated on March 13, 2017, that the AHCA would save $337 billion over the 2017–2026 period, it would also cause the number of uninsured Americans to increase by 14 million in 2018, 21 million in 2020, and 24 million in 2026.^5^ For reference, the number of uninsured non-elderly adults aged 19–65, prior to the implementation of the Patient Protection and Affordable Care Act (also known as PPACA, ACA or Obamacare), hit an all-time high in 2010 at 45 million or 18.3% of the U.S. population, in comparison to an all-time low of 28 million or 10.3% in 2016 under Obamacare.^6^

So, yes, Reps. Mark Meadows and Raúl Labrador are correct when they say that federal law requires everyone to be seen in America’s emergency departments. But what they neglect to mention is that treatment of non-emergent conditions is not and will not be required, and uninsured patients will receive bills for all services rendered. This will return us to the pre-Obamacare era when Americans were sicker and routinely delayed seeking care early for minor problems. With no access to primary care, more Americans will once again need to file for bankruptcy due to lack of health insurance and mounting medical bills. And, in the long run, care for these people will be more expensive to society, when we treat only the emergency when the disease is far advanced.
